# Unveiling the role of cerebellar alterations in the autonomic nervous system: a systematic review of autonomic dysfunction in spinocerebellar ataxias

**DOI:** 10.1007/s00415-023-11993-8

**Published:** 2023-09-26

**Authors:** Nicole Urbini, Libera Siciliano, Giusy Olivito, Maria Leggio

**Affiliations:** 1 Department of Psychology, Sapienza University of Rome, Via dei Marsi 78, 00185 Rome, Italy; 2 Ataxia Laboratory, IRCCS Fondazione Santa Lucia, Via Ardeatina 306-354, 00179 Rome, Italy

**Keywords:** Cerebellum, Autonomic dysfunctions, Cerebellar disorders, Heart rate variability

## Abstract

**Background:**

Autonomic dysfunctions are prevalent in several cerebellar disorders, but they have not been systematically investigated in spinocerebellar ataxias (SCAs). Studies investigating autonomic deficits in SCAs are fragmented, with each one focusing on different autonomic dysfunctions and different SCA subtypes.

**Methods:**

Following the Preferred Reporting Items for Systematic Reviews and Meta-Analyses (PRISMA) statement, we conducted a systematic review of the literature to assess the presence of autonomic dysfunctions in various SCAs. PubMed served as the primary database, and the Rayyan web application was employed for study screening.

**Results:**

We identified 46 articles investigating at least one autonomic function in patients with SCA. The results were analyzed and categorized based on the genetic subtype of SCA, thereby characterizing the specific autonomic deficits associated with each subtype.

**Conclusion:**

This review confirms the presence of autonomic dysfunctions in various genetic subtypes of SCA, underscoring the cerebellum's role in the autonomic nervous system (ANS). It also emphasizes the importance of investigating these functions in clinical practice.

**Supplementary Information:**

The online version contains supplementary material available at 10.1007/s00415-023-11993-8.

## Introduction

Autonomic dysfunctions arise from abnormalities in the autonomic nervous system (ANS), a complex regulatory system responsible for maintaining physiological homeostasis. The ANS consists of the sympathetic and parasympathetic nervous systems, which innervate various visceral organs, each serving distinct functions [1–3]. Through precise control of involuntary physiological processes, the ANS modulates cardiovascular, urinary, gastrointestinal, sexual, sweating, orthostatic, and thermoregulatory functions [1, 2, 4]. Consequently, ANS disruptions can manifest as autonomic dysfunctions in the aforementioned domains, either affecting specific functions or occurring in a more generalized manner, depending on the division involved.

Autonomic dysfunctions can result from diverse factors, including hereditary predispositions, acquired conditions, or even adverse effects of pharmacological treatments [3], or can occur as a result of another disease [5]. Among the pathologies characterized by autonomic dysfunctions [3, 6, 7], multiple system atrophy (MSA) is one of the most common conditions. Specifically, MSA is a neurodegenerative disorder characterized by progressive autonomic failure with extrapyramidal or cerebellar symptoms, which classify it into parkinsonian or cerebellar subtypes [8]. In addition to MSA, other diseases characterized by cerebellar alterations exhibit autonomic dysfunctions, including cerebellar ataxia with neuropathy and vestibular areflexia syndrome (CANVAS) and spinocerebellar ataxias (SCAs), which often copresent with MSA of the cerebellar type [9, 10].

The role of the cerebellum in the ANS is extensively supported by animal and human studies that have identified many connections between brainstem nuclei and higher-order autonomic centers, enabling both direct and indirect influences on autonomic pathways [11–13]. Early studies involving cats and monkeys revealed that the stimulation of different cerebellar areas induced visceral reactions related to urinary, cardiovascular, and respiratory functions [11, 14]. Furthermore, animal studies unveiled direct bidirectional connections between the cerebellum and the hypothalamus, a key area for autonomic control [15, 16]. Notably, the fastigial nucleus and the vermis are closely connected to the hypothalamus [17, 18], and alterations or stimulation of these areas leads to autonomic dysfunctions, including cardiovascular deficits with blood pressure alterations [17, 19, 20]. For a considerable period, the role of the cerebellum in the ANS was primarily explored through animal studies. However, the advent of neuroimaging promoted thorough investigation of the cerebellar–cerebral interplay in regulating body homeostasis. Together with the brainstem and other cortical and subcortical regions, the cerebellum has been recognized as a key region of the central autonomic network (CAN) [21], which regulates ANS functions both at baseline and in response to environmental changes [12, 13]. To date, many studies have confirmed the role of the cerebellum in the modulation of the sympathetic and parasympathetic systems, specifically impacting cardiovascular function through the regulation of blood pressure and heart rate variability (HRV) [12, 13, 22].

The central role of the cerebellum in regulating the ANS and the occurrence of autonomic deficits in cerebellar-related diseases directed our investigation toward the relationship between SCAs of different genetic origins and autonomic dysfunctions. SCAs are rare, genetically inherited neurodegenerative cerebellar disorders that lead to motor, cognitive, and social-affective deficits [23–25]. According to the affected gene, often involving nucleotide triplet expansions, SCAs can be categorized into distinct genetic subtypes, each characterized by a unique onset, symptoms, and progression [26]. The presence of cognitive and social-affective deficits in these pathologies has only recently been recognized, given the purely motor role traditionally attributed to the cerebellum [27–29]. Since it has been established that this brain region also modulates higher-order functions, investigations into the symptomatology of cerebellar pathologies have expanded, including the examination of previously unexplored domains. Among these, autonomic dysfunctions have been identified in various studies and case reports of patients with different genetic SCA subtypes [10, 30–34]. However, the results remain fragmented, and to our knowledge, they have not been systematically reviewed, except for one review that focused on SCA3, a disorder where autonomic dysfunctions appear to be prominent [34]. To overcome this gap, our objective was to systematically review the literature on autonomic dysfunctions in various genetic subtypes of SCA, aiming to discern whether these deficits are a common feature or if some subtypes are more affected than others. Such insights are essential to identifying the currently overlooked autonomic functions in SCAs, which might impact clinical practice and patients' quality of life and autonomy.

## Methods

The present review was conducted according to the Preferred Reporting Items for Systematic Reviews and Meta-analyses (PRISMA) statement 2020 [35], an updated version of the PRISMA statement 2009 [36]. The PubMed database was used, specifically the PubMed Advanced Search Builder. Considering the aim and topics of this review, search terms were selected from Medical Subject Headings (MeSH) and the National Library of Medicine (NLM) controlled vocabulary thesaurus used for PubMed indexing. The last article search was performed on April 4, 2023. The terms were searched in “all fields,” and “humans” and “English language” filters were applied. The search terms included “Spinocerebellar Ataxias” OR “Spinocerebellar Ataxia” OR “Spinocerebellar Atrophies” OR “Spinocerebellar Atrophy” AND “Autonomic Dysfunctions” OR “Autonomic Dysfunction” OR “Autonomic Disorders” OR “Autonomic Disorder” OR “Autonomic Dysfunction, Segmental” OR “Nervous System Diseases, Sympathetic” OR “Nervous System Diseases, Autonomic” OR “Autonomic Nervous System Disorders” OR “Nervous System Diseases, Parasympathetic.” Then, articles were imported into the Rayyan web application, an AI-powered tool for systematic literature reviews [37], where screening and duplicate removal were performed. Two investigators (N.U. and L.S.) independently screened the articles based on the selection criteria and evaluated the titles and abstracts. The inclusion criteria were case reports or experimental studies including adults with a genetic SCA in whom autonomic dysfunctions were investigated or observed. The exclusion criteria were as follows: animal studies; studies not published in English; studies on children; reviews or systematic reviews; study populations without genetically confirmed diagnoses (except for patients with genetically confirmed relatives); study populations with olivopontocerebellar atrophy (OPCA), MSA, or Friedreich's ataxia; and study populations with neurological comorbidities. After the title and abstract inspection, the investigators retrieved the full text of the eligible articles and excluded those that, based on a comprehensive reading, were found to be inconsistent with the selection criteria. In case of disagreement, the investigators discussed to reach consensus and, if necessary, sought the opinion of a third investigator. The full texts and bibliographies of the final included articles were inspected to identify additional articles that met the eligibility criteria. Additional articles were imported into Rayyan for screening.

The following autonomic dysfunctions have been investigated: postural/orthostatic hypotension, postural/orthostatic dizziness, postural/orthostatic syncope, gastrointestinal dysfunctions, urinary dysfunctions, sexual dysfunctions, sweating disorders, cardiac autonomic dysfunctions, thermoregulatory disorders, and heat/cold intolerance. More specifically, gastrointestinal dysfunctions included constipation, diarrhea, nausea, and nonspecified gastrointestinal dysfunctions; urinary dysfunctions included incontinence, dysuria, bladder dysfunctions, voiding difficulties, frequency, urgency, nocturia, retention, and nonspecified urinary disturbances; sweating dysfunctions included hypohidrosis, hyperhidrosis, and nonspecified sweating dysfunctions. When articles did not specify the type of autonomic dysfunction, deficits were reported as “aspecific autonomic dysfunction,” indicating the presence of an autonomic impairment of unspecified nature. In addition, to better characterize the presence of autonomic dysfunctions in genetically different populations, articles were divided based on the specific genetic diagnosis. SCA subtypes for which little evidence was found were combined into a single group to give an overview of the preliminary literature present for less prevalent SCAs. Articles that investigated autonomic functions in more than one type of SCA were cited more than once within each patient group examined. Each article was screened to collect data on genetic diagnosis, experimental or descriptive measures of autonomic dysfunctions, and demographic and clinical characteristics of the study population (males/females, age, illness duration, age of onset, CAG triplet repeats). When available in the text, percentages, means, and standard deviations of the measures of interest were collected. Alternatively, when applicable, these measures were independently calculated from data reported in the articles using an Excel spreadsheet. Additionally, the percentage prevalence of each autonomic dysfunction was calculated for each SCA subtype based on the sum of patients found to be impaired compared with the total sample in which the specific autonomic dysfunction was investigated in the various studies. The calculation was performed via an Excel spreadsheet.

Importantly, in a few papers, autonomic dysfunctions were divided into different subtypes. For example, different subtypes of urinary dysfunctions were sometimes reported, specifying the exact number of patients affected by each subtype. However, since a patient could be affected by multiple subtypes of urinary dysfunction, it was not possible to estimate how many patients in total suffered from overall urinary dysfunction. In cases such as this, the results of the most impaired subcategory have been indicated, but the data have not been included in the prevalence calculation to maintain its truthfulness. Furthermore, in some studies, specific autonomic dysfunctions were analyzed in a smaller number of subjects out of the total study sample. In these cases, prevalence analyses were carried out on the exact number of subjects in which the dysfunction was investigated.

## Results

**Fig. 1:**
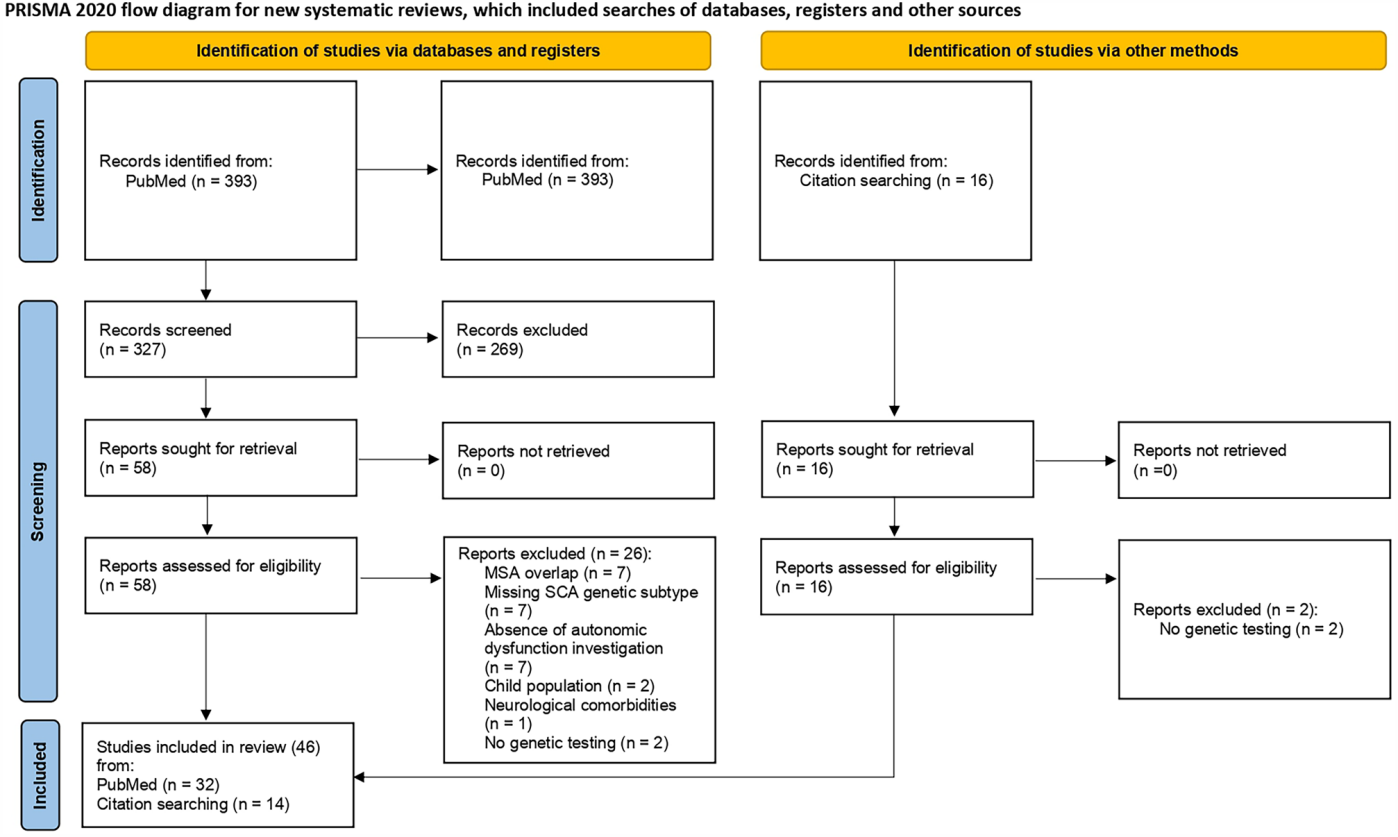
Flow diagram of the methodology and selection of studies following the guidelines of the PRISMA statement 2020 (Page et al., 2021). *MSA* multiple system atrophy; *SCA* spinocerebellar ataxia

The bibliographic search conducted in PubMed identified 393 articles. After Rayyan importation, 66 duplicates were removed, and two investigators (N.U. and L.S.) independently reviewed the studies based on titles and abstracts. This first inspection involved 327 articles, 269 of which were excluded according to the inclusion and exclusion criteria. After full-text reading, the two investigators removed an additional 26 studies, resulting in a total of 32 included articles. The inspection of these studies (text and bibliography) led to the addition and screening of 16 relevant articles, of which two were excluded in accordance with the eligibility criteria. The final analysis involved 46 articles (case reports and original articles) in accordance with the selection criteria. The PRISMA updated flowchart synthesizing the search and screening process is presented in Fig. 1.

In the following paragraphs, the results are reported according to the genetic subtype of SCA. The order of presentation is determined by the number of studies that have investigated each subtype. In each paragraph, autonomic deficits are listed in order of prevalence. Detailed urinary, gastrointestinal, and sweating dysfunctions are shown in Tabs S1, S2, and S3 in the supplementary materials, respectively. The other autonomic dysfunctions were not described in detail as they lack characterization through multiple symptoms or distinct sub-disorders.

### SCA3 and autonomic dysfunctions

The autonomic dysfunctions found in patients with SCA3 are shown in Table 1. Overall, 26 studies investigated or detected the presence of at least one autonomic dysfunction in this population. The most common disorder was heat/cold intolerance, with a prevalence of 52.3% [32, 34, 38]. However, this percentage was calculated from three studies, thus representing a preliminary result that requires further investigation to be confirmed. The second most common dysfunction was cardiac autonomic dysfunction, with a percentage of 44.6% [30, 38–41]; this estimate was based on five studies. Furthermore, three articles detected cardiac autonomic dysfunction as significant in patients compared to control groups [42–44]. Nevertheless, these studies were not included in the calculation due to a lack of specific data on the number of patients with that impairment. In addition, a high prevalence of sweating dysfunctions was observed, which affected 43.9% of the survey population based on 10 studies [32, 34, 38, 39, 41, 42, 45–48]. The most extensively investigated dysfunction was urinary dysfunction, which had a prevalence of 33.5% based on 18 studies [33, 34, 38, 39, 41, 43, 46–57]. This percentage indicates that approximately one-third of the population has urinary problems, and given the large sample size examined, it is likely to be a reliable estimate. Gastrointestinal dysfunctions seem to affect the SCA3 population as well, with a prevalence of 32.4% based on 10 studies [32, 38, 39, 41, 43, 45–48, 58]. Dysfunctions concerning orthostatic issues in SCA3 patients are listed in order of prevalence: orthostatic dizziness (38.9%, 4 studies) [38, 46–48], orthostatic hypotension (25.9%, 7 studies) [32, 38, 41–43, 45, 58], and orthostatic syncope (7.5%, 4 studies) [32, 46–48]. Overall, these findings reveal the presence of an orthostatic alteration that can vary in severity, ranging from dizziness and drastic drops in blood pressure to actual syncope, with the prevalence of symptoms being inversely proportional to the severity of the symptom investigated. Finally, sexual dysfunction showed a prevalence of 25% based on seven studies [34, 38, 39, 45–47, 58].

**Table 1:** Studies reporting autonomic dysfunctions in SCA3 patients

	Sample size	Sex M/F	Age (years)	Age of onset (years)	Duration of illness (years)	CAG repeats	Aspecific autonomic dysfunctions	Orthostatic/postural hypotension	Orthostatic/postural dizziness	Orthostatic/postural syncope	Cold/heat intolerance	Sweating dysfunction	Cardiac autonomic dysfunction	Urinary dysfunction	Sexual dysfunction	Gastrointestinal dysfunction
Watanabe et al.	20	7/13	–	35.4 ± 11.0	11.4 ± 5.4	72.2 ± 3.1	–	–	–	–	–	–	–	11	–	–
Schöls et al.	42	19/23	–	37.5 ± 9.9	10.1 ± 6.1	74.0 ± 3.5	–	–	–	–	–	–	–	8	–	–
Sakajiri et al.	4	1/3	60.3 ± 5.1	–	27.8 ± 10.4	–	–	–	–	–	–	–	–	2	–	–
Soong et al.	25	10/15	42.7 ± 13.9	33.6 ± 11.6	8.3 ± 5.9	73.7 ± 4.9	6	–	–	–	–	–	–	–	–	–
Takiyama et al.	1	1/0	54	–	3	56	–	1	–	–	–	–	–	–	1	1
Yamamoto et al.	4	3/1	48.3 ± 15.0	–	8.0 ± 2.5	–	–	–	–	–	–	–	–	2	–	–
Schöls et al.	60	–	–	36.0 ± 9.0	10.0 ± 6.0	67–82	–	–	–	–	–	–	–	17	–	–
Kazuta et al.	19	6/13	56.0 ± 11.0	–	14.0 ± 6.0	–	–	5	–	–	–	6	+	–	–	–
Yamada et al.	4	1/3	44.5 ± 16.1	18.3 ± 5.4	26.3 ± 17.2	77.5 ± 5.6	–	–	–	–	–	–	–	2	–	–
Uchiyama et al.	3	2/1	70.0 ± 11.5	–	–	–	–	–	–	–	–	1	1	3	1 (2)	3
Gu et al.	3	3/0	49.3 ± 7.8	–	7.0 ± 7.0	56.3 ± 6.1	–	1	–	–	–	2	–	–	1	1
Sakakibara et al.	11	6/5	48.5 ± 12.9	–	9.9 ± 6.2	–	–	–	3	1	–	1	–	11	3 (4)	8
Yeh et al.	15	4/11	40.2 ± 13.2	29.9 ± 10.3	10.3 ± 6.9	76.3 ± 3.9	–	2	7	–	8	11	10 (14)	8	1	3
Maschke et al.	20	–	–	36.0 ± 8.0	9.0 ± 6.0	74.0 ± 4.0	–	–	–	–	–	–	–	9	–	–
Pradhan et al.	5	–	–	–	–	–	–	–	–	–	–	–	3	–	–	–
Schmitz-Hübsch et al.	139	73/66	48.8 ± 11.8	37.1 ± 11.4	11.6 ± 5.9	68.8 ± 4.6	–	–	–	–	–	–	–	63	–	–
Koyama et al.	13	7/6	54.8 ± 13.5	–	11.7 ± 5.2	66.7 ± 5.3	–	–	7	2	–	4	–	3	1 (7)	2
Netravathi et al.	3	2/1	40.0 ± 11.4	36.7 ± 9.5	3.3 ± 2.1	–	–	–	–	–	–	–	3	–	–	–
Asahina et al.	10	4/6	55.0 ± 16.0	–	11.5 ± 8.5	65.9 ± 6.2	–	1	–	–	–	–	+	4	–	2
Musegante et al.	122	–	–	–	–	–	–	–	–	–	–	–	–	17	–	–
Yamanaka et al.	15	7/8	48.9 ± 15.1	–	11.5 ± 7.7	68.6 ± 5.3	–	–	4	0	–	10	–	6	–	6
Takazaki et al.	40	17/23	46.3 ± 12.5	–	8.9 ± 5.2	67.0 ± 5.0	–	10	–	–	–	18	12	15	–	11
Moro et al.	28	11/17	49.7 ± 9.7	36.4 ± 7.8	13.5 ± 7.3	70.0	–	10	–	2	14	11	–	11*	–	8
Jang et al.	26	–	–	–	–	–	–	–	–	–	–	–	–	2	–	–
Tamuli et al.	6	–	–	–	–	–	–	–	–	–	–	+	+	–	–	–
Jin et al.	1	1/0	44	37	7	71	–	–	–	–	1	1	–	1	1	–
Overall prevalence (%)	–	–	–	–	–	–	–	**25.9**	**38.9**	**7.5**	**52.3**	**43.9**	**44.6**	**33.5**	**25.0**	**32.4**

Demographic and clinical data are presented as the mean and standard deviation (age, age of onset, duration of illness, CAG repeats), when available, or as range distribution. Data referring to autonomic dysfunctions represent the number of impaired subjects. The overall prevalence was calculated for autonomic dysfunctions investigated in more than one study and is reported in bold

Numbers in parentheses indicate the number of patients examined for that specific dysfunction, when different from the total sample size of the study. “–” not available; “ + ” dysfunction evidenced with group analysis (SCA3 vs. controls); *M* male; *F* female

*The number of patients presenting with incontinence, the most prevalent urinary dysfunction among the three investigated in this study, was reported. The total number of patients with a general urinary disorder could not be estimated because of the data division (9 nocturia patients, 11 incontinence, 1 urinary retention), and the authors did not specify how many patients had one or more of the reported urinary disorders. Therefore, the study was not included in the analysis of the overall prevalence of this disorder in SCA3 patients

### SCA2 and autonomic dysfunctions

The autonomic dysfunctions found in patients with SCA2 are shown in Table 2. Overall, 18 studies investigated or detected the presence of at least one autonomic dysfunction in this population. Cardiac autonomic dysfunctions were the most prevalent, with a prevalence of 71.6% based on seven studies [30, 40, 59–63]. Furthermore, four studies reported cardiac autonomic dysfunctions in patients with SCA2 when compared to a control group but were not included in the calculation due to lack of data on the number of patients with an impairment [22, 44, 64, 65]. Notably, urinary dysfunctions were found in 37.4% of SCA2 patients, more than one-third of the patients examined [30, 33, 53, 55, 56, 59–61, 63, 64, 66–68]. This dysfunction was the most commonly investigated, providing a result based on 13 studies, which makes the estimate particularly reliable. Additionally, gastrointestinal dysfunctions are also found to have a high prevalence in the SCA2 population, corresponding to 35.3% calculated from six studies [59–61, 64, 66, 67]. Orthostatic hypotension was reported at a prevalence of 28% based on five studies [59–61, 63, 64, 67]. At a smaller percentage, sweating dysfunctions were identified in 23.7% of SCA2 patients based on six studies [30, 40, 59, 63, 64, 67], with one additional study that identified an abnormal sweating response in patients when compared to controls [44]. In addition, sexual dysfunctions were found in 14.2% of SCA2 patients calculated from four studies [59, 60, 64, 67]. Finally, three studies reported aspecific autonomic dysfunctions, with a prevalence of 58.6% [59, 61, 67].

**Table 2:** Studies reporting autonomic dysfunctions in SCA2 patients

	Sample size	Sex M/F	Age (years)	Age of onset (years)	Duration of illness (years)	CAG repeats	Aspecific autonomic dysfunctions	Orthostatic/postural hypotension	Cold/heat intolerance	Sweating dysfunction	Cardiac autonomic dysfunction	Urinary dysfunction	Sexual dysfunction	Gastrointestinal dysfunction
Schöls et al.	11	–	–	32.0 ± 12.0	12.0 ± 8.0	36–52	–	–	–	–	–	4	–	–
Maschke et al.	19	–	–	29.0 ± 11.0	15.0 ± 11.0	38.0 ± 5.0	–	–	–	–	–	5	–	–
Pradhan et al.	6	–	–	–	–	–	–	–	–	1	4	1	–	–
Schmitz-Hübsch et al.	163	75/88	46.3 ± 13.3	34.9 ± 12.7	11.3 ± 6.5	39.3 ± 3.2	–	–	–	–	–	66	–	–
De Joanna et al.	9	6/3	41.6 ± 11.3	30.4 ± 7.4	11.2 ± 5.2	40.5 ± 4.4	8	8	8	2	6	9	2 (6)	7
Netravathi et al.	5	5/0	29.6 ± 15.0	22.3 ± 17.9	7.3 ± 4.8	–	–	–	–	1	5	–	–	–
Montes-Brown et al.	97	–	40.0 ± 10.0	28.1 ± 9.0	11.9 ± 6.0	40.7 ± 3.0	–	–	–	–	61 (74)	38	11	27
Montes-Brown et al.*	48	15/33	37.44 ± 10.6	–	–	36.4 ± 2.6	25	7	–	–	12 (40)	17	–	15
De Rosa et al.	9	6/3	42.0 ± 14.0	30.0 ± 11.0	12.1 ± 7.5	42.4 ± 4.7	–	5	–	1	+	6	2	5
Velázquez-Pérez et al.*	37	13/24	40.1 ± 11.7	54.2 ± 11.8	–	35.7 ± 2.0	–	–	–	–	–	17	–	16
Capozzo et al.	1	1/0	43	43	–	39	1	1	–	1	–	1	1	1
Pedroso et al.	33	9/24	40.7 ± 13.6	32.9 ± 14.0	8.3 ± 6.2	–	–	–	–	–	–	9	–	–
Estévez-Báez et al.	48	24/24	37.4 ± 9.1	27.6 ± 8.0	12.6 ± 5.0	36.8 ± 4.0	–	–	–	–	48	–	–	–
Indelicato et al.	8	5/3	44.3 ± 11.8	31.4 ± 12.5	12.9 ± 5.0	42.0 ± 2.6	–	0	–	3	0	7	–	–
Tamuli et al.	40	30/10	33.3 ± 10.5	27.1 ± 9.7	6.0	42.7 ± 3.9	–	–	–	–	+	–	–	–
Jang et al.	51	–	–	–	–	–	–	–	–	–	–	4	–	–
Tamuli et al.	25	–	–	–	–	–	–	–	–	+	+	–	–	–
Senapati et al.	27	17/10	32.6 ± 14.0	26.0 ± 15.4	6.1 ± 5.7	–	–	–	–	–	+	–	–	–
Overall prevalence (%)	–	–	–	–	–	–	**58.6**	**28.0**	–	**23.7**	**71.6**	**37.4**	**14.2**	**35.3**

Demographic and clinical data are presented as the mean and standard deviation (age, age of onset, duration of illness, CAG repeats), when available, or as range distribution. Data referring to autonomic dysfunctions represent the number of impaired subjects. The overall prevalence was calculated for autonomic dysfunctions investigated in more than one study and is reported in bold

Numbers in parentheses indicate the number of patients examined for that specific dysfunction, when different from the total sample size of the study. “–” not available; “ + ” dysfunction evidenced with group analysis (SCA2 vs. controls); *M* male; *F* female

*Studies on presymptomatic patients

### SCA1 and autonomic dysfunctions

The autonomic dysfunctions found in patients with SCA1 are shown in Table 3. Overall, nine studies investigated or detected the presence of at least one autonomic dysfunction in this population. Cardiac autonomic dysfunctions had a high prevalence in patients with SCA1, namely, 88.2%, based on two studies [30, 40]. In addition, two other experimental studies that were not included in the prevalence calculation due to missing numbers of subjects with this impairment reported cardiac autonomic dysfunctions in SCA1 patients compared to controls [44, 65]. Urinary dysfunctions were the most commonly investigated, with six studies identifying a prevalence of 28% in SCA1 patients [30, 33, 49, 53, 55, 56]. Finally, a single study identified the presence of sweating dysfunctions in the SCA1 population compared to a control group [44].

**Table 3:** Studies reporting autonomic dysfunctions in SCA1 patients

	Sample size	Sex M/F	Age (years)	Age of onset (years)	Duration of illness (years)	CAG repeats	Sweating dysfunction	Cardiac autonomic dysfunction	Urinary dysfunction
Watanabe et al.	20	14/6	–	39.4 ± 10.7	11.9 ± 5.4	47.3 ± 4.4	–	–	3
Schöls et al.	10	–	–	37.0 ± 7.0	7.0 ± 4.0	42–57	–	–	0
Maschke et al.	13	–	–	30.0 ± 9.0	11.0 ± 8.0	49.0 ± 6.0	–	–	5
Pradhan et al.	11	–	–	–	–	–	–	9	1
Schmitz-Hübsch et al.	117	71/46	46.3 ± 12.2	37.0 ± 10.6	9.5 ± 5.5	47.4 ± 5.2	–	–	41
Netravathi et al.	6	5/1	38.7 ± 15.7	32.5 ± 14.9	6.2 ± 4.0	–	–	6	–
Tamuli et al.	31	24/7	35.3 ± 7.8	30.5 ± 7.5	5.0	50.2 ± 6.9	–	+	–
Jang et al.	11	–	–	–	–	–	–	–	1
Tamuli et al.	18	–	–	–	–	–	+	+	–
Overall prevalence (%)	–	–	–	–	–	–	–	**88.2**	**28.0**

Demographic and clinical data are presented as the mean and standard deviation (age, age of onset, duration of illness, CAG repeats), when available, or as range distribution. Data referring to autonomic dysfunctions represent the number of impaired subjects. The overall prevalence was calculated for autonomic dysfunctions investigated in more than one study and is reported in bold

“–” not available; “ + ” dysfunction evidenced with group analysis (SCA1 vs. controls); *M* male; *F* female

### SCA6 and autonomic dysfunctions

The autonomic dysfunctions found in patients with SCA6 are shown in Table 4. Overall, nine studies investigated or detected the presence of at least one autonomic dysfunction in this population. Urinary dysfunctions were the most prevalent in SCA6 patients, with a prevalence of 22.1% based on seven studies [31, 33, 53, 55, 56, 69, 70]. In addition, sexual dysfunctions were identified at a percentage of 6.7% based on evidence from two studies [69, 70]. Regarding orthostatic functions, orthostatic hypotension was found in 5.7% of SCA6 patients, according to three studies [70–72]. Among the other autonomic dysfunctions examined, only one study investigated cardiac autonomic dysfunctions and gastrointestinal dysfunctions in this population [70], and both were absent in the subjects included in the study. Additionally, aspecific autonomic dysfunctions were reported in two studies [69, 70] and were present in 12.5% of the population.

**Table 4:** Studies reporting autonomic dysfunctions in SCA6 patients

	Sample size	Sex M/F	Age (years)	Age of onset (years)	Duration of illness (years)	CAG repeats	Aspecific autonomic dysfunctions	Orthostatic/postural hypotension	Orthostatic/postural dizziness	Cardiac autonomic dysfunction	Urinary dysfunction	Sexual dysfunction	Gastrointestinal dysfunction
Schöls et al.	27	–	–	53.0 ± 11.0	11.0 ± 9.0	22–28	–	–	–	–	2	–	–
Nakagawa et al.	1	0/1	56	56	–	21	–	1	–	–	–	–	–
Azuma et al.	10	6/4	62.0 ± 10.6	–	7.6 ± 4.5	22.4 ± 1.2	–	0	–	–	–	–	–
Maschke et al.	27	–	–	47.0 ± 11.0	13.0 ± 9.0	23.0 ± 1.0	–	–	–	–	4	–	–
Schmitz-Hübsch et al.	107	58/49	64.9 ± 11.0	54.5 ± 10.2	10.4 ± 6.4	22.4 ± 0.9	–	–	–	–	33	–	–
Kim et al.	8	6/2	58.8 ± 11.8	50.9 ± 14.2	7.9 ± 6.2	20–25	4	–	1	–	2	2 (6)	–
Tateno et al.	9	4/5	58.6 ± 10.4	50.3 ± 9.9	8.2 ± 4.0	24.3 ± 2.1	–	–	–	–	5	–	–
Jang et al.	20	–	–	–	–	–	–	–	–	–	3	–	–
Zhang et al.	24	12/8	60.1 ± 12.4	–	11.9	–	0	1	–	0	0	0	0
Overall prevalence (%)	–	–	–	–	–	–	**12.5**	**5.7**	–	–	**22.1**	**6.7**	–

Demographic and clinical data are presented as the mean and standard deviation (age, age of onset, duration of illness, CAG repeats), when available, or as range distribution. Data referring to autonomic dysfunctions represent the number of impaired subjects. The overall prevalence was calculated for autonomic dysfunctions investigated in more than one study and is reported in bold

Numbers in parentheses indicate the number of patients examined for that specific dysfunction, when different from the total sample size of the study. “–” not available; *M* male; *F* female

### Autonomic dysfunctions in multiple SCAs

For most of the other genetic subtypes of SCA, few studies were identified, which did not allow for their individual characterization. Here, we report the results for SCA17, SCA31, SCA7, SCA4, SCA5, SCA8, and SCA10, presented in Table 5. In SCA17, urinary dysfunctions with a prevalence of 46% were identified based on three studies [33, 73, 74]. In addition, one case report described the presence of sexual dysfunctions [74]. In SCA7, urinary dysfunctions had a prevalence of 36.4% based on two studies [33, 55]. In contrast, only two case reports for SCA31 were identified, both of which reported the presence of urinary dysfunctions in the two patients examined; in one patient, sweating dysfunctions and cardiac autonomic dysfunctions were also reported [75, 76]. The results for SCA4, SCA5, and SCA8 originate from the study by Maschke and colleagues (2005), which aimed to investigate urinary dysfunctions in various genetic subtypes. A total of 57% of SCA4, 6% of SCA5, and 46% of SCA8 patients exhibited urinary dysfunction. Finally, one study examined autonomic dysfunctions in SCA10 patients: 28.5% exhibited urinary dysfunctions, 21.4% exhibited cold intolerance and sweating dysfunctions, 14.3% exhibited gastrointestinal dysfunctions, and 3.6% exhibited orthostatic dysfunctions [32].

**Table 5:** Studies reporting autonomic dysfunctions in poorly studied SCA genetic subtypes

Genetic diagnosis		Sample size	Sex M/F	Age (years)	Age of onset (years)	Duration of illness (years)	CAG repeats	Orthostatic/postural hypotension	Orthostatic/postural syncope	Cold/heat intolerance	Sweating dysfunction	Cardiac autonomic dysfunction	Urinary dysfunction	Sexual dysfunction	Gastrointestinal dysfunction
SCA17	De Michele et al.	10	4/6	47.4 ± 9.5	31.4 ± 9.6	16.0 ± 10.8	–	–	–	–	–	–	8	–	–
	Kanai et al.	1	1/0	58	56	2	51	0	–	–	–	–	1	1	–
	Jang et al.	26	–	–	–	–	–	–	–	–	–	–	8	–	–
	(%)	–	–	–	–	–	–	–	–	–	–	–	**46.0**	–	–
SCA7	Maschke et al.	7	–	–	32.0 ± 8.0	8.0 ± 5.0	47.0 ± 4.0	–	–	–	–	–	2	–	–
	Jang et al.	4	–	–	–	–	–	–	–	–	–	–	2	–	–
	(%)	–	–	–	–	–	–	–	–	–	–	–	**36.4**	–	–
SCA31	Sugiyama et al.	1	0/1	73	68	5	**–**	**–**	–	–	–	–	1	**–**	**–**
	Shindo et al.	1	0/1	72	62	10	–	0	–	–	1	1	1	–	–
SCA4	Maschke et al.	14	–	–	36.0 ± 8.0	11.0 ± 10.0	–	–	–	–	–	–	8	–	–
SCA5	Maschke et al.	16	–	–	33.0 ± 10.0	17.0 ± 10.0	–	–	–	–	–	–	1	–	–
SCA8	Maschke et al.	11	–	–	37.0 ± 14.0	15.0 ± 11.0	127.0 ± 46.0	–	–	–	–	–	5	–	–
SCA10	Moro et al.	28	13/15	46.8 ± 15.6	31.7 ± 7.6	15.5 ± 12.0	1990.0	1	1	6	6	–	8*	–	4

Clinical data are presented as the mean and standard deviation (age, age of onset, duration of illness, CAG repeats) when available. Data referring to autonomic dysfunctions represent the number of impaired subjects. The overall prevalence was calculated for autonomic dysfunctions investigated in more than one study and is reported in bold

*The number of patients presenting with incontinence, the most prevalent urinary dysfunction among the three investigated in this study, was reported. The total number of patients with a general urinary disorder could not be estimated because of the data division (8 nocturia patients, 3 incontinence, 0 urinary retention) and the authors not specifying how many patients had one or more of the mentioned urinary disorders

“–” not available; *M* male; *F* female

## Discussion

The primary objective of this systematic review was to investigate the presence of autonomic deficits in patients with diverse genetic subtypes of SCA, aiming to further elucidate the role of the cerebellum within the ANS and to identify potential deficits currently underexplored in clinical practice. The results enabled us to characterize the peculiar autonomic disturbances of each SCA subtype while also assessing preliminary findings for less commonly investigated SCAs. However, despite the abundance of findings available, not all studies investigated the whole body of autonomic dysfunctions, with many focusing on the investigation of specific deficits. As a result, the estimated prevalences provided in this review may be more reliable for certain autonomic dysfunctions, drawing from the results of numerous studies, while others are less reliable. Subsequently, the results obtained for different genetic subtypes will be discussed, followed by a comparison between different SCAs to explore the potential involvement of the cerebellum in determining the observed autonomic deficits.

Autonomic dysfunctions have been extensively investigated in SCA3 patients, leading us to identify 26 relevant studies (see Table 1 for references to the specific studies). According to the results obtained, the autonomic dysfunctions with more robustly estimated prevalences include urinary dysfunction, gastrointestinal dysfunction, sweating dysfunction, orthostatic hypotension, and sexual dysfunction. Among these, sweating dysfunction demonstrated the highest prevalence, followed by urinary dysfunction and gastrointestinal dysfunction, each affecting approximately one-third of SCA3 patients. Additionally, sexual dysfunction and orthostatic hypotension were apparent in approximately one-quarter of the population. These findings are highly relevant, indicating that a significant proportion of SCA3 patients experience diverse, frequently coexisting autonomic deficits. Moreover, although they have been less extensively investigated, other characteristic impairments have been identified in this population, including cardiac autonomic dysfunction, which occurred in nearly half of patients. This prevalence, as elucidated in the results, is also corroborated by studies confirming the presence of this deficit in SCA3 patients when compared to control subjects. In contrast, findings related to heat/cold intolerance and other orthostatic deficits are of a preliminary nature and are supported by only a few studies. However, according to the existing evidence, heat/cold intolerance may be present in over half of the SCA3 population, while orthostatic deficits, such as orthostatic dizziness and orthostatic syncope, have a higher prevalence when the disease is more severe, with a higher prevalence for dizziness following a change of orthostatic position compared to actual syncope. To date, only one other study has attempted to estimate the prevalence of autonomic dysfunctions in SCA3 patients [34]. However, this case report, which included a review of SCA3 autonomic deficits, was based on a smaller number of studies. When compared to the findings of the present review, the prevalences reported by Jin and colleagues (2022) appear to be discrepant. These discrepancies may arise from differences in the analysis sample or the categorization approach. For instance, the authors distinguished various urinary dysfunctions and gastrointestinal dysfunctions, possibly influencing the prevalence outcomes. Additionally, the lack of criteria used in their analysis further hinders direct comparisons. Overall, including the results of Jin and colleagues (2022), patients with SCA3 manifest numerous autonomic changes that affect different domains and may present individually or in a more generalized manner.

Autonomic dysfunction in SCA2 patients was investigated in 18 studies (see Table 2 for study details). The calculated prevalences indicate that cardiac autonomic dysfunction appears to be predominant in this population, with a remarkably high percentage. A substantial number of studies were devoted to investigating this function, and additional studies supported the presence of this deficit in SCA2 when compared to control groups. Indeed, the identification of cardiovascular deficits in the majority of enrolled patients provides a reliable estimation of these dysfunctions, warranting greater attention in clinical practice. Similarly, urinary dysfunction and gastrointestinal dysfunction were identified in over one-third of SCA2 patients, with reliable prevalence estimates. Other autonomic dysfunctions, such as orthostatic hypotension and sweating dysfunctions, appear to be present in approximately one-quarter of the population, despite the limited number of studies supporting this calculation. Last, sexual dysfunction seems to be less frequent in SCA2 patients, although further investigation is needed. Overall, the results concerning autonomic deficits in SCA2 are interesting, particularly the clear identification of cardiac autonomic dysfunction, which could represent a distinctive deficit of this genetic subtype. Moreover, similar to SCA3, urinary dysfunction and gastrointestinal dysfunction seem to be frequently present, representing a potential common trait among SCAs.

In the SCA1 patient population, estimates of autonomic dysfunctions were derived from 9 studies (see Table 3 for study details). Autonomic dysfunctions have been less investigated in this population, resulting in the estimation of prevalences for only two deficits. Despite this limitation and the need for cautious interpretation, cardiac autonomic dysfunction was identified in a remarkably high percentage of SCA1 patients. The results strongly indicate the presence of this dysfunction in patients and are further supported by other studies comparing SCA1 patients with control subjects. Clinically, it is crucial to consider cardiovascular changes, as they could serve as a prominent feature of this disease. Additionally, urinary dysfunction has also been the subject of investigation in several studies, although at a lower prevalence of approximately one-third of the population.

In comparison to the genetic subtypes discussed above, SCA6 is an exception. Although the findings are based on nine studies (see Table 4 for a detailed overview), autonomic dysfunction appears to be minimal in this disorder. Specifically, a very low prevalence or absence of deficits was found for sexual dysfunction, orthostatic hypotension, cardiac autonomic dysfunction, and gastrointestinal dysfunction. Urinary dysfunctions are the only autonomic dysfunctions reported in approximately one-fifth of the population, yet they are still lower than in other SCAs. These results reaffirm that urinary dysfunction may be a trait potentially common to all SCAs, as well as the sole trait present in the SCA6 population, which is overall marked by limited autonomic impairments. These findings align with the literature, which characterizes SCA6 as a genetic subtype with late onset, slow progression, fewer deficits, and an unchanged life expectancy compared to other SCAs [77, 78].

The results for all the other investigated genetic subtypes of SCA (see Table 5 for specific study details) were fragmented, thereby preventing a specific characterization of autonomic dysfunction unique to each SCA. However, the results of the included studies further underscore the presence of urinary dysfunction as a common trait across all SCAs, being observed in SCA17, SCA31, SCA7, SCA4, SCA8, SCA10, and with a very low prevalence in SCA5. Other autonomic functions have only been reported in a few case reports or in a single genetic subtype based on very limited studies. Consequently, the results obtained will not be discussed in detail.

Overall, the characterization of different genetic subtypes of SCA revealed common alterations across all disorders and distinctive autonomic dysfunctions in certain SCAs. In particular, patients with SCA3 and SCA2 exhibited deficits in multiple autonomic functions, suggesting that these two pathologies might be the most affected, with more generalized damage at the level of the ANS. Notably, these were also the pathologies with more studies available, which could indicate either the actual predominant presence of autonomic deficits in these populations or merely the greater focus on autonomic dysfunctions in SCA2 and SCA3 patients compared to other SCAs. These findings align with the reported alterations in the autonomic ganglia and fiber tracts in both subtypes [54, 79]. Moreover, in SCA3, a reduction in the number of neurons in the intermediolateral nucleus of the lateral gray column in the spinal cord has been implicated in autonomic failure [51].

Analyzing the results, urinary dysfunction appears to be a common autonomic dysfunction across all SCAs, presenting with varying prevalence in each genetic subtype, even in SCA6, in which other autonomic domains are absent. A potential explanation lies in a SPECT study conducted on patients with MSA, revealing a decrease in cerebellar vermis activation during urinary storage and micturition compared to controls, highlighting cerebellar involvement in urinary functions [80]. Additionally, as reported in the review by [81], functional neuroimaging studies indicate a cerebellar role in urinary storage by modulating bladder and sphincter activity. Given the known link between the cerebellum and ANS [12, 13] and the alterations at the level of the sympathetic and parasympathetic systems responsible for urinary deficits [82, 83], it is reasonable to hypothesize that cerebellar alterations in patients with SCA prevent the precise regulation of urinary autonomic functions, contributing to the observed disorders.

Cardiac autonomic dysfunctions and gastrointestinal dysfunctions are the two other disorders frequently observed in many of the investigated genetic subtypes. According to De Joanna and colleagues (2008), the alterations in autonomic nuclei and autonomic fiber tracts found by Gierga and colleagues (2005) in SCA2 patients could explain the cardiac autonomic and gastrointestinal disorders reported in this patient population. The disturbances in cardiac autonomic functions could arise from a lack of cerebellar control, supported by recent evidence that correlates the HRV response with the activation of the right cerebellar cortex in patients with different SCA subtypes [44]. Furthermore, electrophysiological data suggest that cerebello-hypothalamic connections regulate key areas involved in feeding behavior, such as the gastric vagal afferents, thus providing an explanation for some of the gastrointestinal symptoms observed in SCAs [84].

In conclusion, based on the identified autonomic dysfunctions and the supporting evidence for the role of the cerebellum in these dysfunctions, it can be asserted that SCAs frequently present with autonomic deficits that may arise from cerebellar degeneration. These deficits warrant further investigation to facilitate their identification and treatment in clinical practice.

## Limitations and future directions

To our knowledge, this systematic review represents the first investigation into autonomic dysfunctions in patients with different genetic subtypes of SCA. However, it is essential to acknowledge some limitations. Despite employing strict inclusion and exclusion criteria, the inclusion of both case reports and studies with varying methodologies might introduce bias in the estimated prevalences. However, given the limited number of studies investigating these dysfunctions in patients with SCAs, imposing stricter criteria could result in missing important results. Additionally, the data provided by the authors in the studies did not allow further classification of patients according to sex, precluding the assessment of potential differences in autonomic deficits between men and women in the various investigated SCAs.

Considerations pertaining to extracerebellar alterations in SCAs warrant thorough examination. Particularly, SCAs like SCA2, SCA3, and SCA1 exhibit extensive damage not only within the cerebellum but also across the brainstem, pons, basal ganglia, thalamus, and midbrain [78]. Notably, even SCA6 patients, recognized primarily for cerebellar pathologies, display diffuse degeneration within these aforementioned regions [78]. Given the autonomic functions governed by these areas, which are part of the ANS [21, 85], it is plausible that the identified autonomic dysfunctions stem from this widespread alteration rather than solely from cerebellar involvement. Nonetheless, considering the acknowledged modulatory capacity of the cerebellum within motor, cognitive, and socio-affective circuits [86, 87], as well as its connections linking to ANS-related areas [12, 13, 21], it becomes a viable hypothesis that the pronounced degeneration of the cerebellum in SCAs significantly influences the autonomic functions in SCA patients. However, the precise role of the cerebellum within the extensive networks interconnected in autonomic regulation necessitates further exploration through future research.

Notwithstanding the aforementioned limitations, this review marks a significant step toward identifying the potential role of the cerebellum in the onset of autonomic deficits, a domain previously overlooked in this population and one that will need further investigation.

With regard to the cerebellum's role in autonomic deficits, further exploration is warranted, especially in relation to cardiac autonomic dysfunction. The majority of the studies that assessed cardiac autonomic dysfunction in various SCAs focused on measures of HRV, an index of cardiac parasympathetic control [88]. However, the recognized role of HRV in detecting cardiac autonomic changes only scratches the surface of its potential. Recent research has revealed connections between HRV and behavioral regulation, emotion regulation, emotion recognition, and social skills, including theory of mind [89–92]. Given the acknowledged role of the cerebellum as a modulator in these domains, these functions have been extensively studied and found to be impaired in SCAs [29, 93–95]. Consequently, the cardiac autonomic deficits found in this systematic review could also be linked to the emotional deficits present in patients with SCA, particularly considering the connections the cerebellum has with the limbic system, which plays a role in modulating autonomic reactions in emotional processing [93, 96]. This evidence underscores the potential role that the cerebellum might play in cardiac autonomic dysregulation in various SCAs, where it has been identified as one of the predominant autonomic deficits. Furthermore, the lack of cerebellar control over autonomic functions could result in difficulties in dampening autonomic reactivity to emotional stimuli, leading to the emotional and social deficits observed, which have seldom been attributed to autonomic dysregulation until now. Hence, investigating the close connection between the cerebellum, cardiac autonomic deficits, HRV, and emotion regulation through further studies is crucial. This exploration may also pave the way for the development of noninvasive cerebellar stimulation treatments aimed at ameliorating these deficits in patients, especially considering the positive outcomes obtained thus far with such approaches [97–100].

### Supplementary Information

Below is the link to the electronic supplementary material.

Supplementary file1 (DOCX 49 KB)

## Data Availability

The data that support the results of this systematic review are available from the corresponding author upon reasonable request.
