# Supplementary material for: Unveiling the role of cerebellar alterations in the autonomic nervous system: a systematic review of autonomic dysfunction in spinocerebellar ataxias
Source: J Neurol. 2023 Sep 26;270(12):5756–72. doi: 10.1007/s00415-023-11993-8 (PMC10632228; doi:10.1007/s00415-023-11993-8)
Supplement: Supplementary file 1 — Supplementary file1 (DOCX 49 KB) [file 415_2023_11993_MOESM1_ESM.docx]

|  |  | Sample size | Incontinence | Dysuria | Bladder dysfunctions | Voiding difficulties | Frequency | Urgency | Nocturia | Retention | Non-specified urinary disturbances |
| --- | --- | --- | --- | --- | --- | --- | --- | --- | --- | --- | --- |
| SCA3 |  |  |  |  |  |  |  |  |  |  |  |
|  | Watanabe et al. | 20 | – | – | – | – | – | – | – | – | 11 |
|  | Schöls et al. | 42 | 8 | – | – | – | – | – | – | – | – |
|  | Sakajiri et al. | 4 | – | 2 | – | – | – | – | – | – | – |
|  | Yamamoto et al. | 4 | – | – | – | – | – | – | – | – | 2 |
|  | Schöls et al. | 60 | 17 | – | – | – | – | – | – | – | – |
|  | Yamada et al. | 4 | – | – | – | – | – | – | – | – | 2 |
|  | Uchiyama et al. | 3 | – | 3 | – | – | – | – | – | – | – |
|  | Sakakibara et al. | 11 | 4 | – | – | 7 | – | 3 | 4 | – | – |
|  | Yeh et al. | 15 | 2 | – | – | – | – | – | 8 | – | – |
|  | Maschke et al. | 20 | 9 | – | – | – | – | – | – | – | – |
|  | Schmitz-Hübsch et al. | 139 | – | – | – | – | – | – | – | – | 63 |
|  | Koyama et al. | 13 | – | – | – | – | – | – | – | – | 3 |
|  | Asahina et al. | 10 | – | – | – | – | – | – | – | – | 4 |
| SCA3 | Musegante et al. | 17 | 9 | – | 3 | 4 | 8 | 15 | 6 | 9 | – |
|  | Yamanaka et al. | 15 | – | – | – | 3 | – | – | – | 4 | – |
|  | Takazaki et al. | 40 | – | – | – | – | – | – | – | – | 16 |
|  | Moro et al. | 28 | 11 | – | – | – | – | – | 9 | 1 | – |
|  | Jang et al. | 26 | – | – | – | – | – | – | – | – | 20 |
|  | Jin et al. | 1 | – | – | – | – | – | – | 1 | 1 | – |
| SCA2 | Schöls et al. | 11 | 4 | – | – | – | – | – | – | – | – |
|  | Maschke et al. | 19 | 5 | – | – | – | – | – | – | – | – |
|  | Pradhan et al. | 6 | – | – | – | – | 1 | – | – | – | – |
|  | Schmitz-Hübsch et al. | 163 | – | – | – | – | – | – | – | – | 66 |
|  | De Joanna et al. | 9 | – | – | – | – | – | – | – | – | 9 |
|  | Montes-Brown et al. | 97 | – | – | – | – | – | – | – | – | 38 |
|  | Montes-Brown et al. | 48 | – | – | – | – | – | – | – | – | 17 |
|  | De Rosa et al. | 9 | – | – | – | – | – | – | – | – | 6 |
|  | Velázquez-Pérez et al. | 37 | – | – | – | – | – | – | – | – | 17 |
|  | Capozzo et al. | 1 | 1 | – | – | – | – | – | – | – | – |
|  | Pedroso et al. | 33 | – | – | – | – | – | – | – | – | 9 |
|  | Indelicato et al. | 8 | – | – | – | – | – | – | – | – | 7 |
|  | Jang et al. | 51 | – | – | – | – | – | – | – | – | 4 |
| SCA1 | Watanabe et al. | 20 | – | – | – | – | – | – | – | – | 3 |
|  | Schöls et al. | 10 | 0 | – | – | – | – | – | – | – | – |
|  | Maschke et al. | 13 | 5 | – | – | – | – | – | – | – | – |
|  | Pradhan et al. | 11 | – | – | – | – | 1 | – | – | – | – |
|  | Schmitz-Hübsch et al. | 117 | – | – | – | – | – | – | – | – | 41 |
|  | Jang et al. | 11 | – | – | – | – | – | – | – | – | 1 |
| SCA6 | Schöls et al. | 27 | 2 | – | – | – | – | – | – | – | – |
|  | Maschke et al. | 27 | 4 | – | – | – | – | – | – | – | – |
|  | Schmitz-Hübsch et al. | 107 | – | – | – | – | – | – | – | – | 33 |
|  | Kim et al. | 8 | – | – | – | – | 2 | – | – | – | – |
|  | Tateno et al. | 9 | 3 | – | – | 1 | 3 | 2 | 1 | 0 | 5 |
|  | Jang et al. | 20 | – | – | – | – | – | – | – | – | 3 |
|  | Zhang et al. | 24 | – | – | – | – | – | – | – | – | 0 |
| SCA17 | De Michele et al. | 10 | 8 | – | – | – | – | – | – | – | – |
|  | Kanai et al. | 1 | 1 | – | – | – | – | – | – | – | – |
|  | Jang et al. | 26 | – | – | – | – | – | – | – | – | 8 |
|  |  |  |  |  |  |  |  |  |  |  |  |
| SCA7 | Maschke et al. | 7 | 2 | – | – | – | – | – | – | – | – |
|  | Jang et al. | 4 | – | – | – | – | – | – | – | – | 2 |
| SCA31 | Sugiyama et al. | 1 | – | – | – | – | – | – | – | 1 | – |
|  | Shindo et al. | 1 | 1 | – | – | – | – | – | – | – | – |
| SCA4 | Maschke et al. | 14 | 8 | – | – | – | – | – | – | – | – |
| SCA5 | Maschke et al. | 16 | 1 | – | – | – | – | – | – | – | – |
| SCA8 | Maschke et al. | 11 | 5 | – | – | – | – | – | – | – | – |
| SCA10 | Moro et al. | 28 | 3 | – | – | – | – | – | 8 | 0 | – |

**Table S1.** Detailed urinary dysfunctions reported in studies investigating this specific domain in various SCAs. Data represent the number of impaired subjects in that specific urinary dysfunction. “–”: not available.

|  |  | Sample size | Constipation | Diarrhea | Nausea | Non-specified gastrointestinal dysfunctions |
| --- | --- | --- | --- | --- | --- | --- |
| SCA3 |  |  |  |  |  |  |
|  | Takiyama et al. | 1 | 1 | – | – | – |
|  | Uchiyama et al. | 3 | 1 | – | – | – |
|  | Gu et al. | 3 | 1 | – | – | – |
|  | Sakakibara et al. | 11 | 8 | – | – | – |
|  | Yeh et al. | 15 | 2 | 1 | 2 | – |
|  | Koyama et al. | 13 | 2 | – | – | – |
|  | Asahina et al. | 10 | 2 | – | – | – |
|  | Yamanaka et al. | 15 | 6 | – | – | – |
|  | Takazaki et al. | 40 | – | – | – | 12 |
|  | Moro et al. | 28 | 8 | 0 | – | – |
| SCA2 | De Joanna et al. | 9 | – | – | – | 7 |
|  | Montes-Brown et al. | 97 | – | – | – | 27 |
|  | Montes-Brown et al. | 48 | – | – | – | 15 |
| SCA2 | De Rosa et al. | 9 | – | – | – | 5 |
|  | Velázquez-Pérez et al. | 37 | – | – | – | 16 |
|  | Capozzo et al. | 1 | 1 | – | 1 | – |
| SCA6 | Zhang et al. | 24 | – | – | – | 0 |
| SCA10 | Moro et al. | 28 | 4 | – | 0 | – |

**Table S2.** Detailed gastrointestinal dysfunctions reported in studies investigating this specific domain in various SCAs. Data represent the number of impaired subjects in that specific gastrointestinal dysfunction. “–”: not available.

|  |  | Sample size | Hypohidrosis | Hyperhidrosis | Non-specified sweating syfunction |
| --- | --- | --- | --- | --- | --- |
|  |  |  |  |  |  |
| SCA3 | Kazuta et al. | 19 | – | – | 6 |
|  | Uchiyama et al. | 3 | 1 | – | – |
|  | Gu et al. | 3 | – | – | 2 |
|  | Sakakibara et al. | 11 | – | – | 1 |
|  | Yeh et al. | 15 | – | – | 11 |
|  | Koyama et al. | 13 | 4 | – | – |
|  | Yamanaka et al. | 15 | 5 | – | – |
|  | Takazaki et al. | 40 | – | – | 18 |
|  | Moro et al. | 28 | – | 11 | – |
|  | Jin et al. | 1 | – | 1 | – |
| SCA2 | Pradhan et al. | 6 | – | 1 | – |
|  | De Joanna et al. | 9 | – | – | 2 |
|  | Netravathi et al. | 5 | – | 1 | – |
|  | De Rosa et al. | 9 | – | – | 1 |
| SCA2 | Capozzo et al. | 1 | – | 1 | – |
|  | Indelicato et al. | 8 | – | – | 3 |
| SCA31 | Shindo et al. | 1 | – | – | 1 |
| SCA10 | Moro et al. | 28 | – | 6 | – |

**Table S3.** Detailed sweating dysfunctions reported in studies investigating this specific domain in various SCAs. Data represent the number of impaired subjects in that specific sweating dysfunction. “–”: not available.
